# From Concept to Clinic: A Scoping Review of Robotics and Virtual Reality-Assisted Rehabilitation Protocols in Neurological and Orthopaedic Conditions

**DOI:** 10.7759/cureus.109222

**Published:** 2026-05-19

**Authors:** Mugdha D Oberoi, Diya Barmecha, Summaiya Zareen Shaikh, Anjali S Puntambekar

**Affiliations:** 1 Electrotherapy and Electrodiagnosis, K.J. Somaiya College of Physiotherapy, Mumbai, IND; 2 Biomedical Sciences, University College London (UCL), London, GBR; 3 Neuro Physiotherapy, The SIA College of Health Sciences, College of Physiotherapy, Thane, IND; 4 Musculoskeletal Physiotherapy, The SIA College of Health Sciences, College of Physiotherapy, Thane, IND

**Keywords:** gamification in healthcare, neurological conditions, orthopedic conditions, physiotherapy intervention, robotics, virtual reality simulation, vr assisted rehabilitation

## Abstract

Robotics and virtual reality (VR) have shifted physiotherapy away from traditional hands-on treatment toward more technologically driven rehabilitation. These tools, ranging from exoskeletons and gait trainers to immersive VR environments, introduced a new way of approaching neuro-muscular recovery. Robotic systems provided precise movement data and real-time feedback, making sessions more personalised and consistent. VR and gamification placed patients in interactive environments that improved engagement and helped reduce pain perception. Together, these technologies offered a more dynamic approach to rehabilitation while also enabling better progress tracking for both clinicians and patients.

However, this shift was not without its challenges. Much of the research surrounding these technologies focused on device efficacy rather than providing workable clinical protocols. This left clinicians without clear guidance, contributing to apprehension around adoption. The absence of structured training programmes and regulatory frameworks only added to this uncertainty, making it difficult to implement these systems alongside existing physiotherapy practice.

This scoping review aimed to address these gaps by mapping existing rehabilitation protocols that incorporated robotic and VR technologies. Session frequency and duration, patient selection criteria, and integration with conventional therapies were all examined. The theoretical foundations of each protocol were considered alongside reported clinical outcomes. The overall aim was to support a smoother transition toward a hybrid model of care, one that combines the precision of technology with the clinical expertise of physiotherapists, ultimately improving both functional outcomes and patient engagement in rehabilitation settings.

## Introduction and background

The development of robotics and virtual reality (VR) orchestrated a paradigm shift from traditional physiotherapy to more technologically advanced interventions. This transition marked a step forward into a novel era of neuro-muscular rehabilitation, including initial assessment, recovery, and strengthening of the joints following any strain, injury, or general strengthening [1]. Robotic rehabilitation incorporated the use of sensors, exoskeletons, and gait trainers [2], which provoked precise and accurate movement data to the system, which helped enhance motor recovery. Robotic rehabilitation also enabled real-time monitoring [3] and continuous, encouraging feedback; this made the sessions with patients more personalised and, hence, more effective. Whereas with VR and gamification [4], patients were immersed in a virtual environment where their exercises were performed interactively, thereby enhancing patient retention and promoting multisensory feedback. This gamification approach helped reduce the perception of pain [5]. Furthermore, a technologically advanced rehabilitation system helped track progress data for both clinicians and patients.

However, with a sudden change in the style of treatment, there were efficiency and protocol issues that arose. Although there were many theoretical and practical benefits with this type of approach, it was still experimental research [6]. Many studies done on robotic rehabilitation systems and VR systems focused on the efficacy of the device but did not provide a detailed protocol that clinicians could adopt. This left the development of standardised protocols up to each clinician, leading to apprehension [7] in using such a new system. There was also a lack of comprehensive training programmes and regulatory guidelines [8]. Having a system in place to have robotic rehabilitation along with traditional physiotherapy practices was required. Integrating both practices, offering a hybrid approach, ensured that the technological benefits were supported by the clinical expertise of physiotherapists.

In this scoping review, these gaps were addressed by systematically mapping existing rehabilitation protocols that used robotic and VR technology. The objective was to identify protocol details, examine their theoretical base and then evaluate the reported outcomes. This included analysing hard parameters such as the frequency and duration of the session, the selection of patient criteria, and the integration with conventional therapies. Finally, the review aimed to ease the transition from traditional practice to a more hybrid or technologically dependent approach with the goal of improving both functional outcomes and patient engagement under rehabilitation settings.

Methods

Framework

This scoping review adopted the methodological framework proposed by Arksey and O’Malley (2005). In addition to this, it was enhanced with recommendations from the PRISMA-ScR (Preferred Reporting Items for Systematic Reviews and Meta-Analyses extension for Scoping Reviews) checklist [9]. The PRISMA-ScR checklist was used to guide transparent and comprehensive reporting of the methodology, ensuring alignment with international standards, thereby enhancing the review’s credibility.

Inclusion and Exclusion Criteria

The inclusion criteria were adults (≥18 years) undergoing rehabilitation for neurological or musculoskeletal impairments. The intervention involved the use of robotics and/or VR in rehabilitation protocols. Studies conducted in clinical or community rehabilitation settings were included. Eligible sources were peer-reviewed research articles, clinical trials, and protocol papers published in English between 2010 and 2024.

The exclusion criteria included studies involving healthy participants, individuals with primary cardiovascular or respiratory conditions, and animal studies. Studies that did not involve robotics or VR in rehabilitation protocols were excluded. Studies conducted exclusively in experimental or laboratory-based settings without a clinical rehabilitation focus were also excluded. Reviews, editorials, commentaries, conference abstracts, and grey literature were not considered. Studies not published in English or published prior to 2010 were also excluded.

Information Sources and Search Strategy

In order to search for the studies in the initial phases, electronic databases such as PubMed, PEDro, and Scopus were used. Studies that fit the inclusion criteria, with keywords combined terms related to rehabilitation ("physical therapy" or "rehabilitation"), technology ("robotics" or "virtual reality"), and protocols ("intervention" or "protocol"). Specifically, the search strategy combined these using "physical therapy" AND "robotics" AND "protocol". Microsoft Excel (Microsoft Corporation, Redmond, Washington) was utilised, and a filter was set for language and studies published after 2010. After removal of duplicates, titles and abstracts were screened independently by two reviewers. The articles were then assessed independently by both reviewers using predefined eligibility criteria. Any disagreements were resolved through discussion or, when necessary, consultation with a third reviewer. All potentially relevant articles were reviewed against the inclusion criteria. Data that were extracted included title, source, context, population, device and type, protocol details, outcome measures, and key findings.

**Figure 1:**
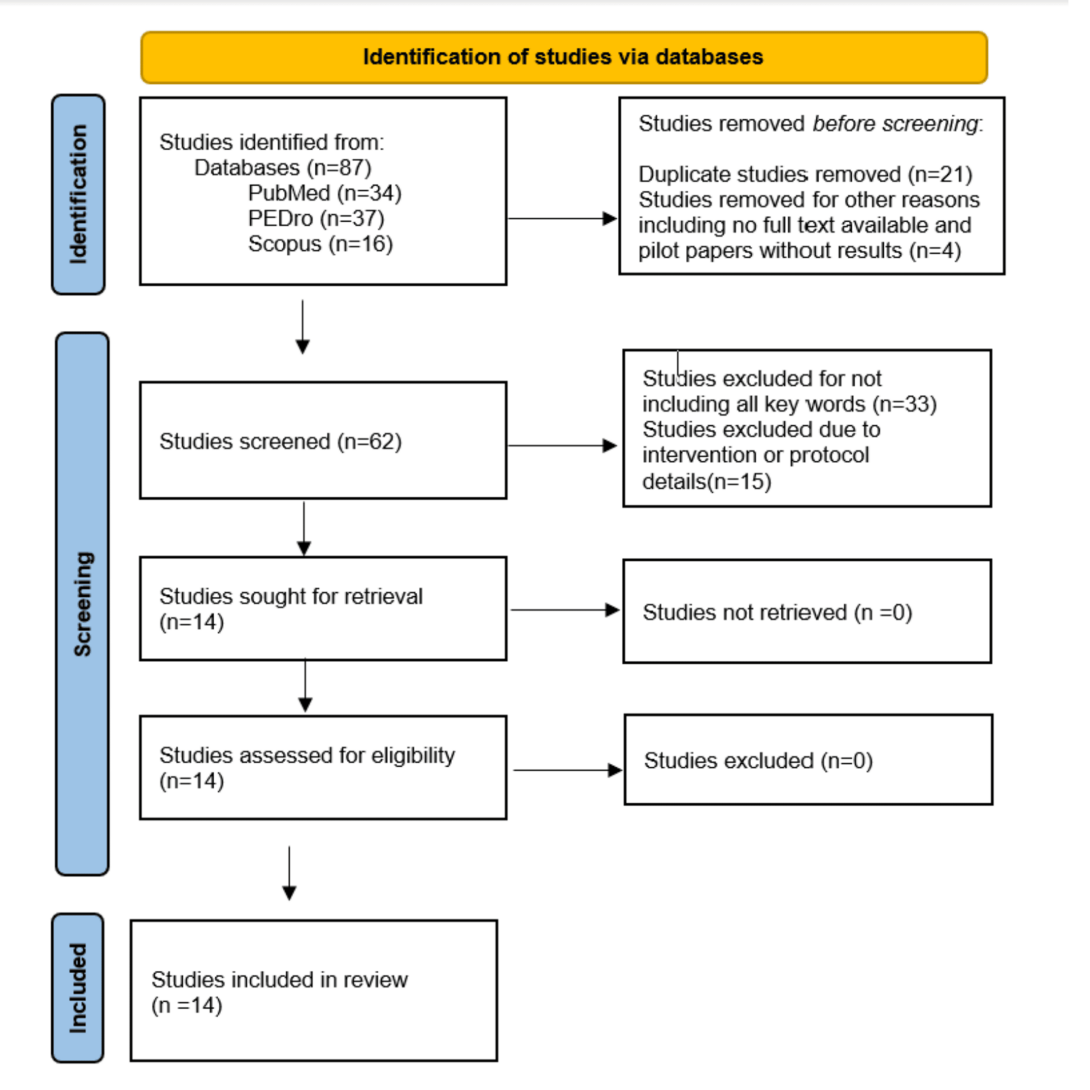
Article Selection Flowchart

A total of 87 articles were initially screened from all sources. However, based on the inclusion criteria, only 14 were selected. These 14 articles were carefully reviewed to ensure they met all criteria of inclusivity and adhered to a standard protocol. Additionally, the selected articles were organised in a clear and systematic manner to facilitate analysis. Specifically, six articles were sourced from PubMed [10-15], six from PEDro [16-21], and two from Scopus [22,23]. Although the initial searches were conducted using these databases, the full texts were obtained either through direct download or by accessing them via other journal platforms. An article selection flowchart is presented in Figure 1.

Overview of Studies

A total of 87 records were identified through the search process. Following screening and full-text eligibility assessment, 14 studies were included in this scoping review. The included studies were conducted across multiple regions: three in Asia, seven in Europe, and four in North America. More specifically, the studies originated from Japan (1) [10], Spain (2) [11,13], Canada (2) [12,20], Italy (3) [16,19,22], the United States (2) [17,18], Taiwan (1) [21], South Korea (1) [23], Portugal (1) [14], and Poland (1) [15]. There was great geographical diversity in the selection of the studies.

The populations selected from the different studies ranged from a minimum of 27 [14] to a maximum of 143 [21] patients. These were either patients with orthopaedic or neurological conditions. From the selected literature, 10 studies investigated interventions for stroke survivors, of which three focused specifically on individuals with hemiplegia [10,17,23]. Two studies involved patients with multiple sclerosis [11,13], while two studies targeted orthopaedic conditions, specifically shoulder pathology [19] and hip arthroplasty [22]. These studies could have been either peer-reviewed research articles or clinical trials. Following these inclusion criteria, of the 14 studies selected, three were observational studies [12,14,15], while the remaining 11 were clinical trials, including pilot trials and randomised controlled trials.

Several studies incorporated neuroplasticity concepts [10,16,18,23] to support the use of intensive and repetitive training, particularly for neurological populations. A few studies referred to the International Classification of Functioning, Disability and Health (ICF) framework to align intervention targets with broader activity goals [12,14,21]. However, none of the studies mentioned technology adoption theories, despite the clear relevance of such frameworks for understanding how patients and clinicians interact with new rehabilitation technologies.

Regarding study design, 12 of the 14 included studies employed a two-group structure, consisting of a control group and an intervention group. Two studies used a three-group design to compare a control group with two distinct intervention groups; one tested different combinations of therapies [15], and the other tested variations of the same intervention approach [10]. Follow-up periods and outcome measures varied across studies but were commonly motor function, gait, or patient-reported measures of motivation.

Summary of Key Findings

Across the included studies, protocols for robotic devices showed considerable variation, with durations ranging from 2 to 12 weeks, individual sessions lasting 30 to 90 minutes, and a frequency of two to five sessions per week. By contrast, VR interventions generally use shorter sessions, lasting 20 to 40 minutes, typically two to three times weekly for four to eight weeks, which is likely to reduce fatigue associated with VR tasks. Interventions commonly targeted balance, gait training, and mobility integrated with gamification and real-time feedback. These features helped to enhance patient satisfaction and engagement.

The target domain for eight of the studies was the upper limb [10,11,13,16-20] while four focused on gait and postural stability [12,14,15,23], one on lower limb mobility [22], and one on multiple limbs [21]. Outcome measures varied across studies, reflecting the diverse aims of the interventions. Six studies assessed multiple functional outcomes [11,13,15,18,19,21], such as range of motion, gait speed, and upper-limb dexterity, while eight studies focused on a single primary outcome with supporting secondary measures. For upper limb rehabilitation, the Fugl-Meyer Assessment was the most commonly used tool since three studies [10,16,20] used it. Other primary outcomes included the Functional Ambulation Category [12], Action Research Arm Test [17], Hip Disability Index [22], and gait speed [14,23]. Several studies also used qualitative methods, such as semi-structured interviews and focus groups, to gather insights on patient experiences and satisfaction with VR or robotic therapy. At times, these interviews were also supported by qualitative assessments such as the Fatigue Severity Scale, Katz Index, and McGill Pain Questionnaire.

When comparing results, both quantitative and qualitative findings were mapped. Of the 14 studies, eight reported quantitative improvements, but six showed statistically significant effects [13,15,16,18,21,23]. Out of these eight, one study did not examine qualitative outcomes. Meanwhile, the other six studies found qualitative improvements, such as higher motivation and satisfaction, but four of these did not show matching quantitative gains [10-12,19], and one of these did not assess quantitative changes at all [12]. Across studies, at least one outcome showed some improvement. Though the nature and significance of these findings varied considerably, the results should be interpreted with caution, given the methodological heterogeneity. One study specifically noted that although improvements were observed, they were not statistically significant, emphasising the need for further work to refine and test detailed intervention plans [17]. The six studies that showed statistically significant qualitative improvements were all combining physical movements with cognitive tasks. These cognitive tasks were either motor imagery or interactive gaming. Amplified therapy effectiveness was achieved due to this. Furthermore, in most of the studies that achieved improvement, the devices had the capability to adjust assistive and resistive modes based on patient capability in real time.

Several of the studies used objective biomechanical or kinetic measures, such as motion capture systems and surface EMG, alongside clinical scales such as the Fugl-Meyer and Berg Balance Scale.

Even with so many studies, there were still doubts on the optimal protocol intensity and duration for maximising benefit over traditional therapy. Most studies used a two-group design, so the effects of different training or session frequencies remained unclear. For this reason, using a three-group design in future research could help with protocol parameters. Even the study that used the three-group design did not differ by protocol but instead by therapy, using a hybrid approach combining robotics with conventional methods. In addition, some studies did not correctly describe the technical aspects of the devices used for the study, such as sensors or hardware specifications. This made it harder to replicate and translate, as not every device was available to every clinic. A table of quantitative results has been summarised below to support the above claims (Table 1).

**Table 1:** Summary of Quantitative Data RCT: Randomised Controlled Trial; FMA-UE: Fugl-Meyer Assessment Upper Extremity; BBT: Box and Block Test; ARAT: Action Research Arm Test; WMFT: Wolf Motor Function Test; PENN: Penn Spasm Frequency Scale; HOOS JR: Hip Disability and Osteoarthritis Outcome Score Junior

Study Details	Methodology & Sample Characteristics	Intervention Characteristics	Protocol & Functional Domain	Outcome Measures & Findings
Takebayashi et al., 2022 [10]	RCT; Stroke with hemiplegia; n=129	ReoGo-J; Robotics; 3 groups	10 weeks; 60 min/session; Upper limb	FMA-UE; Not significant
Marcos-Anton et al., 2023 [11]	RCT; Multiple sclerosis; n=40	MYO Armband®; Robotics/sEMG; 2 groups	8 weeks; 60 min/session; Upper limb	Goniometry, grip strength, BBT; p=0.004 (supination, grip strength)
Louie et al., 2020 [12]	Study protocol; Stroke; n=40	Exoskeleton; Robotics; 2 groups	8 weeks; 60 min/session; 5×/wk; Gait	Functional Ambulation Category; Qualitative improvement only
Cuesta-Gomez et al., 2020 [13]	RCT; Multiple sclerosis; n=28	Leap Motion Controller; VR/digital; 2 groups	10 weeks; 60 min/session; Upper limb	Coordination, speed, UL dexterity; p<0.05
Rodrigues et al., 2022 [14]	Research article; Stroke; n=27	PhysioSensing; VR/digital; 2 groups	40 weeks; 45 min/session; 2×/wk; Gait	Fall risk, gait speed; −7.9% fall risk, NS (between groups)
Stepien et al., 2025 [15]	Research article; Stroke; n=66	Erigo®Pro + Riablo™; Robotics; 3 groups	2 weeks; Gait	BBS, static balance; p<0.05 (TA tension), trend for balance
Frisoli et al., 2022 [16]	RCT; Stroke; n=26	L-EXOS; Robotics; 2 groups	6 weeks; 45 min/session; Upper limb	FMA-UE, BBT; p<0.01 (both), p<0.05 (robot superior proximally)
Wolf et al., 2015 [17]	RCT; Stroke with hemiplegia; n=99	Hand Mentor Pro; Robotics; 2 groups	8–12 weeks; 3 hrs/session; 5×/wk; Upper limb	ARAT, WMFT; Not significant
Brokaw et al., 2014 [18]	Pilot crossover trial; Stroke; n=12	ARMin III + HandSOME; Robotics; 2 groups	1 month/block (~12 hrs); Upper limb	FMA, ARAT, BBT; p<0.05
Rizzato et al., 2023 [19]	RCT; Shoulder pathology; n=22	Playball®; VR/digital; 2 groups	10 sessions; 40 min/session; Upper limb	PENN score, pain, strength; p<0.01 (pain), p<0.05 (others)
Hernandez et al., 2022 [20]	RCT; Stroke; n=51	VR + telerehab; VR/digital; 2 groups	4 weeks; 2 sessions; Upper limb	FMA-UE; Not significant
LIn et al., 2021 [21]	RCT; Stroke; n=143	Kinect VR; VR/digital; 2 groups	5 days; 15 min ×2/day; Multi-domain	Strength, mood, function; p<0.05
Fascio et al., 2022 [22]	RCT; Hip arthroplasty; n=43	VRRS; VR/digital; 2 groups	15 days post-op; Lower limb	HOOS JR; NS (group), p<0.05 (time)
Lee et al., 2025 [23]	Clinical trial; Stroke with hemiplegia; n=45	GEMS-A; Robotics; 2 groups	Single session; Gait	Gait speed; p<0.05

## Review

Summary of context

This scoping review aimed to map the landscape of technologically assisted rehabilitation. It enabled the tracing of their journey from conceptual development to application in clinical practice. Technologically assisted rehabilitation used VR, exoskeletons, and robotics to enhance personalisation and engagement [24] in therapy for individuals recovering from neurological or musculoskeletal conditions. VR helped create an immersive and interactive environment, removing feelings of pain and helping to replicate real-life tasks. This allowed patients to practise functional movements in a safe and motivating setting. Meanwhile, robotics offered controlled and repeatable movement. This could be assistive or resistance-based [25] depending on the patient. Robotics and VR were both used alongside an exoskeleton, which helped guide movements [26]. Together, these innovations reflected the growing shift from traditional therapist-dependent rehabilitation to a more technologically assisted style of patient care where the patient's needs were recognised and acted upon [27].

The studies included in this literature review showed a diverse range of devices and protocols. Studies investigating upper limb rehabilitation used various devices, including ReoGo-J, Myo armband, ARMin, PlayBall, and Hand Mentor Pro. Even though all these devices aimed to support upper-limb rehabilitation, they differed in their mechanical design and sensor technologies. ReoGo-J and ARMin were exoskeletons, also called end-effector devices [28]. These devices helped provide guided and repetitive arm movements. They used position encoders and torque sensors to monitor joint angles and the force applied. Meanwhile, the Hand Mentor Pro used pneumatic actuators and pressure sensors to monitor resistance. The PlayBall [19] was a sensory therapy ball that used pressure sensors and gyroscopes to detect grip force and fine motor control. The Myo armband [29] was a wearable that used surface electromyography sensors to detect muscle activation and capture motion data. Even though they all aimed to activate muscle engagement and motor learning, they were different in their sensors and biomechanical data. However, they shared core similarities in their real-time motion tracking and sensor integration.

Similarly, for lower limb rehabilitation focused on gait training, various mechanical systems could be used. One such type was a wearable robotic exoskeleton that provided powered assistance to joints while walking; this was the GEMS-A [23] and was used in a study for stroke patients. It used motion sensors to monitor stride length and walking speed in real time. PhysioSensing [15] was a pressure-sensitive balance platform that used pressure sensors to study weight shifting, and the Erigo Pro Table [30] was a tilt table that used gradual tilting of patients to control movement patterns. For all devices, whether for the upper or lower limb, they shared similar key features. These included the integration of real-time data, feedback mechanisms, and repetitive training [7]. These were the same principles of traditional rehabilitation, just delivered by sensors instead of the therapist.

VR interventions utilised immersive and non-immersive systems [31], often combined with gaming elements to enhance motivation. Immersive VR used head-mounted displays and, at times, full-body tracking sensors to fully engage the user within the three-dimensional virtual space [32]. Non-immersive VR relies on standard screens alongside motion capture cameras or other wearable sensors to detect movements and translate them into interactive tasks on the screen. Of the studies screened, four used VR [13,20-22].

Overall, this review highlighted how technological innovations in VR and robotics were moving beyond conceptual prototypes [33] to become integrated, adaptable components of modern rehabilitation practice.

Limitations

Across the studies, many ongoing challenges and issues related to limited generalisability were identified [12,13,16,18]. This mainly occurred due to small sample sizes that would not represent broad patient populations or varied contexts. Many studies even went further to acknowledge the possibility that participants of the trial might have, in the same duration of the trial, sought additional care at other healthcare facilities [10,14,17,18,23] or conducted some at-home exercises introducing unaccounted for and confounding factors that would have influenced outcome changes. Due to this, there was no ability to isolate the effects of the robotic intervention. Furthermore, due to shorter follow-up periods [11,19,21] and outcome measurement constraints [20,22,23], it was difficult to assess whether the improvements and observed benefits were sustained due to the robotic or VR intervention alone.

In addition, several studies exhibited methodological weaknesses, such as a lack of patient blinding [10,12,22] and, in one case, an absence of intergroup comparisons [20]. These things reduced the reliability of the evidence presented. One of the studies solely relied on parametric tests [15] despite the presence of complex individual factors when statistically analysing the recovery trajectories. This may have led to an oversimplification of the statistical interpretation of the results.

These limitations showcased clear areas for improvement in future research and clinical applications of technology rehabilitation. There was a need for broader sampling strategies, longer follow-ups, and varied protocols to better understand the effectiveness of these technologies. Even when discussing translation into clinical settings, methodological gaps left practical implications. For example, the limited generalisation across patient groups made it difficult for therapists to know which patient groups would benefit the most. Overall, these gaps underscored the need for more detailed study designs and protocols to advance these technologies from concept to clinic.

## Conclusions

The journey from concept to clinic for technology-assisted rehabilitation remained fragmented and needed to be enhanced through evidence-based protocol standardisation. In addition, further implementation research was essential to realise their full impact. The review highlighted how these technology-driven innovations had helped bridge the gap between conventional therapist-led interventions and personalised and technologically enhanced care. The studies reviewed in this demonstrated that protocols combining repetitive and task-specific training with real-time feedback and gamification could produce measurable improvements in functional outcomes. Additionally, all studies with patient feedback consistently showed improvement in patient motivation and engagement.

However, these findings also highlighted persistent gaps and challenges that needed to be addressed to translate these interventions into widespread clinical practice. Most of these studies lacked standardised protocols and clear reporting of device specifications, which limited generalisation and reproducibility. Due to small sample sizes, short follow-up duration, and varied outcome measures, the strength of the evidence base was decreased. By addressing these challenges, researchers and clinicians could help ensure the theoretical and practical benefits of robotics and VR, giving patients greater personalisation and enhanced engagement. In order to advance this field, future research was expected to focus on developing standardised protocols that would help integrate robots and VR with conventional therapy in a hybrid approach. Having three-arm studies enabled the testing of multiple protocols simultaneously. There was a need for well-designed studies with longer follow-up periods and multi-modal outcome measures. In conclusion, a clearer evidence base would help clinicians and therapists confidently adopt technology-assisted rehabilitation to improve patient motivation and outcomes.
